# Diabetic retinopathy with extensively large area of capillary non-perfusion: characteristics and treatment outcomes

**DOI:** 10.1186/s12886-022-02508-6

**Published:** 2022-07-04

**Authors:** Zijing Huang, Kunliang Qiu, Jingsheng Yi, Hongjie Lin, Dezhi Zheng, Dingguo Huang, Guihua Zhang, Haoyu Chen, Jianlong Zheng, Yifan Wang, Danqi Fang, Weiqi Chen

**Affiliations:** Joint Shantou International Eye Center of Shantou University and the Chinese University of Hong Kong, 69 North Dongxia Rd, Shantou, 515041 Guangdong China

**Keywords:** Diabetic retinopathy, Large area, Capillary non-perfusion, Laser photocoagulation

## Abstract

**Background:**

Capillary non-perfusion is an important characteristic for diabetic retinopathy (DR) indicating microvascular damage and ischemia. Data on the description and treatment outcomes of DR with large area of non-perfusion are lacking to date. We aim to describe the characteristics and treatment outcomes in a series of patients with DR who presented extensively large area of capillary non-perfusion (LACNP).

**Methods:**

Fundus fluorescein angiograms from medical charts in patients diagnosed with DR between Jan 2017 and Dec 2019 were retrospectively reviewed. Clinical data in eyes with LACNP including imaging and laboratory findings at the first presentation were analyzed. The LACNP was defined as over 70% area of capillary non-perfusion throughout the whole image retina. The mean follow-up duration was 12.4 ± 16.7 months. Follow-up data including extensive pan-retinal photocoagulation and surgical intervention and treatment outcomes were evaluated.

**Results:**

A total of 43 eyes in 24 patients with LACNP were included, accounting for 3.3% of DR populations in the same period. The overall percentage of non-perfusion area was 79.1 ± 8.1%. All patients received proper control of diabetes and hypertension, and extensive pan-retinal laser photocoagulation. During the follow-up periods, 20 eyes (46.5%) developed severe neovascular complications, of which 15 eyes (34.9%) underwent vitrectomy and/or anti-glaucoma surgeries. Conservative therapies including glycemic control and supplemental laser photocoagulation were conducted in 23 eyes (53.5%) without neovascular complications. In the final follow-up, best corrected visual acuity improved or maintained stable in 19 eyes (44.2%) while deteriorated in 24 eyes (55.8%).

**Conclusions:**

The presence of LACNP is the hallmark of advanced DR and often indicates a poor visual outcome, although aggressive treatments may slow DR progression and maintain central vision for some time.

**Supplementary Information:**

The online version contains supplementary material available at 10.1186/s12886-022-02508-6.

## Background

Diabetic retinopathy (DR) is a common microvascular complication of diabetes and the leading cause of blindness in working-age population worldwide [1]. The microvascular damage mediated by high levels of blood glucose induces microvascular block and capillary non-perfusion (CNP) in the retina, as well as exudation and hemorrhage spots, called non-proliferative diabetic retinopathy (NPDR). The ischemia triggers the release of vascular endothelial growth factor (VEGF) and other proangiogenic factors, leading to neovascularization, which is the hallmark of proliferative diabetic retinopathy (PDR) [2–4].

The area of CNP is an important characteristic for DR that serves as a potential predictive biomarker for NPDR severity as well as PDR activity [5, 6]. Visualization of retinal capillaries and quantification of area of CNP are available using fundus fluorescein angiography (FFA). The ischemic index, defined as the percentage area of ischemic retina calculated based on total visible retina [7], was reported to be around 25-50% [8, 9]. However, we noted in clinical work that a subgroup of DR patients suffers from an large area of capillary non-perfusion (LACNP) where retinal microperfusion can hardly be seen throughout the whole visible retina except for the macular region. Some eyes even further develop macular non-perfusion, which is impressing and thought-provoking. To our knowledge, little has been reported about the characteristics, treatment and prognosis in this subgroup. In this study, we aimed to explore these by retrospectively reviewing patients with DR and LACNP in our eye center.

## Materials and methods

### Subjects

This retrospective interventional case series was conducted at the Joint Shantou International Eye Center of Shantou University and The Chinese University of Hong Kong, Guangdong, China. The study adhered to the guidelines of the Declaration of Helsinki, approved by the Ethic Committee of Joint Shantou International Eye Center of Shantou University and The Chinese University of Hong Kong, and was registered on the Chinese Clinical Trial Registry (ChiCTR 2000033098). Informed consent was exempt as it was a retrospective study. Consecutive patients from Jan 2017 to Dec 2019 who met the following criteria were included:

1) diagnosed with DR according to the guidelines of diabetic retinopathy by the American Academy of Ophthalmology [10]; 2) FFA exam showed a large area of CNP in the whole retina except the macular region. The large area of CNP is defined as over 70% of the area of capillary non-perfusion throughout the whole visible retina, with or without macular non-perfusion. 3) had not preiously received any anti-DR treatment, including laser photocoagulation, anti-VEGF injections, vitrectomy, and others.

Exclusion criteria included:

1) had a history of congenital retinal vasculopathy; 2) combined with other vision-threatening or retinal vascular disorders, such as glaucoma, retinitis pigmentosa; retinal vein occlusion, retinal artery occlusion, etc. 3) Fluorescein angiograms showed poor quality due to severe media opacity, hemorrhage or severe proliferation.

### Data acquisition

A retrospective review of fundus fluorescein angiograms in patients diagnosed with DR between Jan 2017 and Dec 2019 was performed. Eyes showing LACNP at their first presentation were selected. Clinical data at baseline, treatment and follow-ups were reviewed. Ocular examination included best corrected visual acuity, intraocular pressure, wide-field scanning laser ophthalmoscope (SLO), optical coherence tomography (OCT), and fundus fluorescein angiography (FFA). Medical records were reviewed for age, gender, diabetes including the course, levels of fasting blood glucose and Hemoglobin A 1c (HbA1c), systemic hypertension, complete blood count, blood biochemistry, and renal function tests. Neovascular complications at the time of fluorescein angiogram and at follow-up periods, including retinal neovascularization, neovascular glaucoma, vitreous hemorrhage, and tractional retinal detachment, were analyzed.

### Quantification for nonperfusion area based on FFA imaging

FFA Images were digitally archived and reviewed from the medical records in JSIEC. Images were obtained with a scanning laser ophthalmoscope after standard intravenous infusion of 5 mL of 10% sodium fluorescein. Each single image enables 55° field of the retina. Mid-peripheral and peripheral imaging was done with appropriate eye movement. Photoshop CC 2015 (San Jose, CA, USA) was used to synthetize the series of images with the same overlapped scene to enable a large wide viewing angle of around 130° of the posterior pole. Image rotation and adjustment of brightness/contrast and were allowed when doing the splicing.

The area of CNP was assessed by two independent reviewers. Briefly, the area of CNP was encircled using the area measurement function of Photoshop and divided by the total visible area. The reviewers were allowed to enhance the images by using the smoothing function and optimize function; by adjusting contrast, brightness, and gamma. Vascular leakage was not considered as CNP. Any inconsistence in the judgment of CNP between the two reviewers was further determined by a supervisor. In addition, angiographic macular ischemia, defined as a destroyed foveal avascular zone and a 30 percent increase in the foveal avascular area, was also evaluated.

### Laser photocoagulation

All the eyes with LACNP received extensive pan-retinal laser photocoagulation [Supra Scan; Quantel Medical.] with hope to induce regression of new vessels and prevent neovascular complications. Laser photocoagulation was performed using single spot pattern laser (532 nm wave length, 100-400μm spot size, 120-320mW power, and 200ms duration). The distribution of photocoagulation started from the boundaries between the ischemic and non-ischemic regions at the posterior pole and extended to the very peripheral retina, as close as possible to the ora serrata. Small light spots (100~200μm diameter) were applied in the posterior hole of retina whereas big spots (200~ 400μm diameter) were used in the peripheral region. The individual burns were applied throughout the non-perfusion area, separated by approximately half of burn diameter. The photocoagulation was divided into several sessions to reduce the risk of adverse effects such as exudative retinal detachment, angle closure, and macular edema. Macular grid laser treatment (single spot pattern 532nm wave length, 100μm spot size, 100mW power, and 100ms duration) was performed in eyes with persistent macular edema.

### Surgical intervention and assessment of prognosis

Surgical procedures were performed in eyes developing neovascular complications during the follow-up periods. Intravitreal injection of anti-VEGF agents was performed in eyes with clinically significant macular edema, neovascular glaucoma, or as the pretreatment before PPV surgery for complicated PDR. For eyes with severe vitreous hemorrhage or tractional retinal detachment, standard 23-gauge PPV with (*n*=10) or without (*n*=1) phacoemulsification was performed based on the presence or absence of cataract. For eyes with refractory neovascular glaucoma, glaucoma valve implantation was applied (*n*=4).

The visual outcomes were evaluated. Worsening of DR include a deterioration of BCVA with one or more in the following during the follow-up periods: extension of LACNP to the macular region, tractional retinal detachment, new onset of vitreous hemorrhage, neovascular glaucoma, and macular atrophy. An improved or maintained BCVA with no such findings was considered as stabilization of DR and LACNP. BCVA changes from baseline are defined as improved with an increase of 1 or more ETDRS line, or worsened if there was a decrease of 1 line or more. Otherwise, it was considered stable.

### Statistical analysis

The area of CNP was measured manually using the GraphPad Prism 6 software (GraphPad Software, San Diego, CA). Statistical analysis on levels of blood glucose, blood pressure, HbA1c, creatinine, urea nitrogen and other laboratory findings was performed using SPSS 18.0 software (SPSS Inc, Chicago, USA). Quantitative data were present as mean ± standard deviation. BCVA was converted to logMAR before statistical analysis.

## Results

A total of 43 eyes in 24 patients (14 female and 10 male, 56.0 ± 9.0 years old) with LACNP were reviewed, which accounted for 3.3% of all the angiograms from our DR database during the same period. The baseline data in this analysis are shown in Table 1. Of note, 19 patients had both eyes presenting with LACNP. Unilateral LACNP was observed in 5 patients due to imaging unavailable with severe vitreous hemorrhage (*n*=4) or absence of LACNP in the contralateral eye (*n*=1). The duration of presenting symptoms of diabetes was 7.3 years on average in all patients with the mean levels of fasting blood glucose 13.6 mmol/L and HbA1c 8.1%. In addition, nine of the patients (37.5%) had a history of hypertension. Their controlled blood pressure was 153.8/91.3 mmHg on average before FFA examination. In addition, impaired renal function, presenting with elevation of creatinine and urea nitrogen, was found in 75.0% of the patients.

**Table 1:** Patient demographics at the time of fluorescence fundus angiography

Characteristics	Number or levels (range)^c^
Gender	
Male	10
Female	14
Average age (years)	56.0 ± 9.0 (35~71)
Course of DM^a^ (years)	7.3 ± 5.6 (1~25)
Hypertension (mmHg)	153.8 ± 11.9 / 91.3 ± 6.4, *n*=9
Fasting blood glucose (mmol/L)	13.6 ± 4.7 (6.6~21.7)
HbA1c^b^ (%)	8.1 ± 1.7 (5.5~12.7)
Renal function	
Creatinine (μmol/L)	122.3 ± 57.1 (52~280)
Urea nitrogen (mmol/L)	9.9 ± 3.6 (5.3~20.2)
Hemoglobin (g/L)	125.0 ± 11.0 (107~147)
Albumin (g/L)	42.5 ± 9.6 (29.2~77.5)

^a^DM diabetes mellitus

^b^HbA1c hemoglobin A1c

^c^Data are mean ± SD (range)

**Fig. 1:**
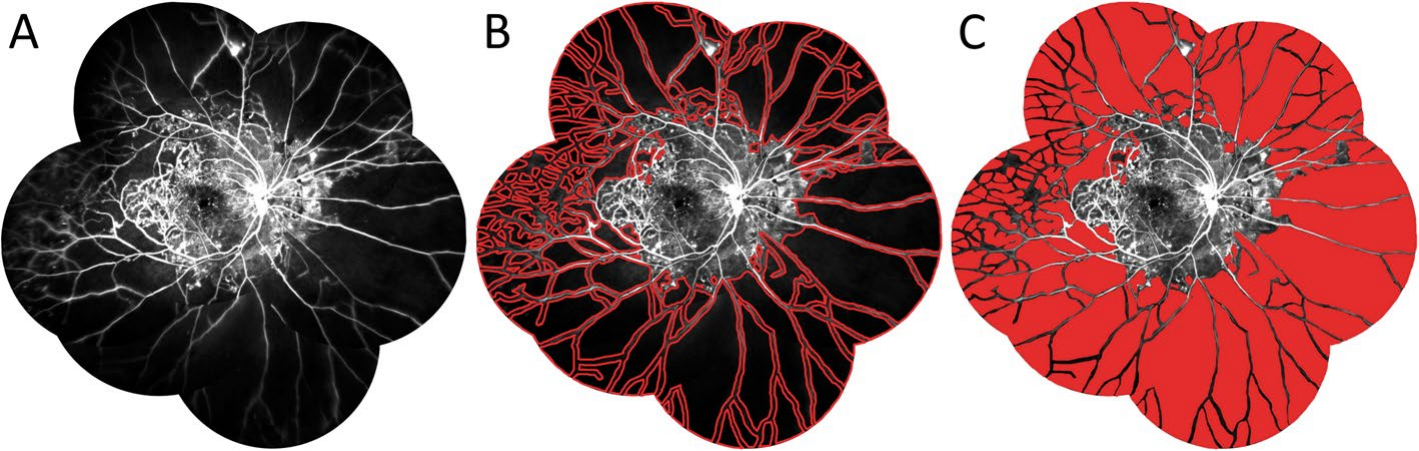
Diagrams showing measurement of area of capillary non-perfusion (CNP) in diabetic retinopathy. **A** Angiographic images of a patient with large area of retinal capillary non-perfusion except the macular region. **B**, **C** The area of CNP was measured by manually circling the CNP area (**B**) and filling it with color (**C**) using the Image-Pro Plus 6.0 software. The measurement was performed by two dependent researchers and an average was taken

**Fig. 2:**
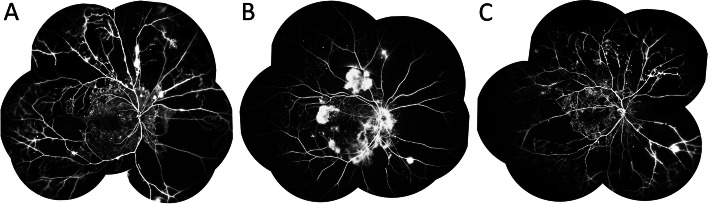
Representative images of excessively large area of capillary non-perfusion (LACNP) in diabetic retinopathy. **A**-**C** Angiographic images of 3 eyes with LACNP. Neovascularization and vascular leakage were found in the peripheral (**A**) and mid-peripheral area (**B**). Local ischemia of the macula was noted (**C**)

All patients underwent FFA exam after proper control of diabetes and hypertension. The overall percentage of CNP area in all included eyes was 79.1 ± 8.1%. Representative FFA images on LACNP were shown in Figs. 1 and 2. In addition, retinal neovascularization was found in all subjects, which located either on or within one disc diameter of the optic disc (34.9%), elsewhere (44.2%), or both (20.9%), indicating the presence of proliferative DR. In addition, angiographic macular ischemia was identified in 58.1% of the eyes.

Fundus photography was performed in all subjects through which retinal hemorrhage and exudation were seen in 15 eyes (34.9%) and 14 eyes (32.6%), respectively. Neovascular vessels or tufts accounted for 30.2% of the subjects. In addition, pre-retinal proliferative membrane at the posterior pole suggesting an advanced stage of DR of the retina was noted in 10 eyes (23.3%).

High quality OCT was obtained in 33 eyes of the subjects. Sixteen of these eyes demonstrated cystoid macular edema with an average macular foveal thickness of 434.4 ± 239.3μm while 9 eyes had diffused edema with an averaged thickness of 291.4 ± 138.8μm. In addition, 29 eyes (87.9%) demonstrated evidence of disorganization of retinal inner layers (DRIL). Interruption of the ellipsoid zone (EZ, also known as IS/OS) and retinal cysts were noted in 57.6% and 48.5% of the eyes, respectively (Table 2).

**Table 2:** Imaging characteristics of eyes with LACNP

Characteristics	Number (percentage)
Fundus fluorescence angiography	
LACNP	43 (100%)
Area of non-perfusion	79.1 ± 8.1%
Neovascularization	43 (100%)
NVD	15 (34.9%)
NVE	19 (44.2%)
NVD+NVE	9 (20.9%)
Macular ischemia	25 (58.1%)
Fundus photography	
Hemorrhage	15 (34.9%)
Exudation	14 (32.6%)
Neovascularization	13 (30.2%)
Proliferative membrane	10 (23.3%)
Optical coherence tomography, *n*=33	
Macular edema	
CME	16 (48.5%)
Diffused edema	9 (27.3%)
DRIL	29 (87.9%)
Interruption of the EZ	19 (57.6%)
Retinal cysts	16 (48.5%)

*Abbreviations*: *LACNP* large area of capillary non-perfusion, *NVD* new vessels on or within one disc diameter of the optic disc, *NVE* new vessels elsewhere, *CME* cystoid macular edema, *DRIL* disorganization of retinal inner layers, *EC* ellipsoid zone

With proper control of blood pressure and glucose, all the patients underwent extensive pan-retinal laser photocoagulation in several sessions, with a total of 1798.0 ± 724.9 burns per eye. Five eyes received combined macular grid laser treatment due to persistent macular edema. Intravitreal injection of anti-VEGF agents was performed in 17 eyes (39.5%) due to macular edema (*n*=2), neovascular glaucoma (*n*=4), or as the pretreatment before pars plana vitrectomy (PPV) for complicated PDR (*n*=11).

**Fig. 3:**
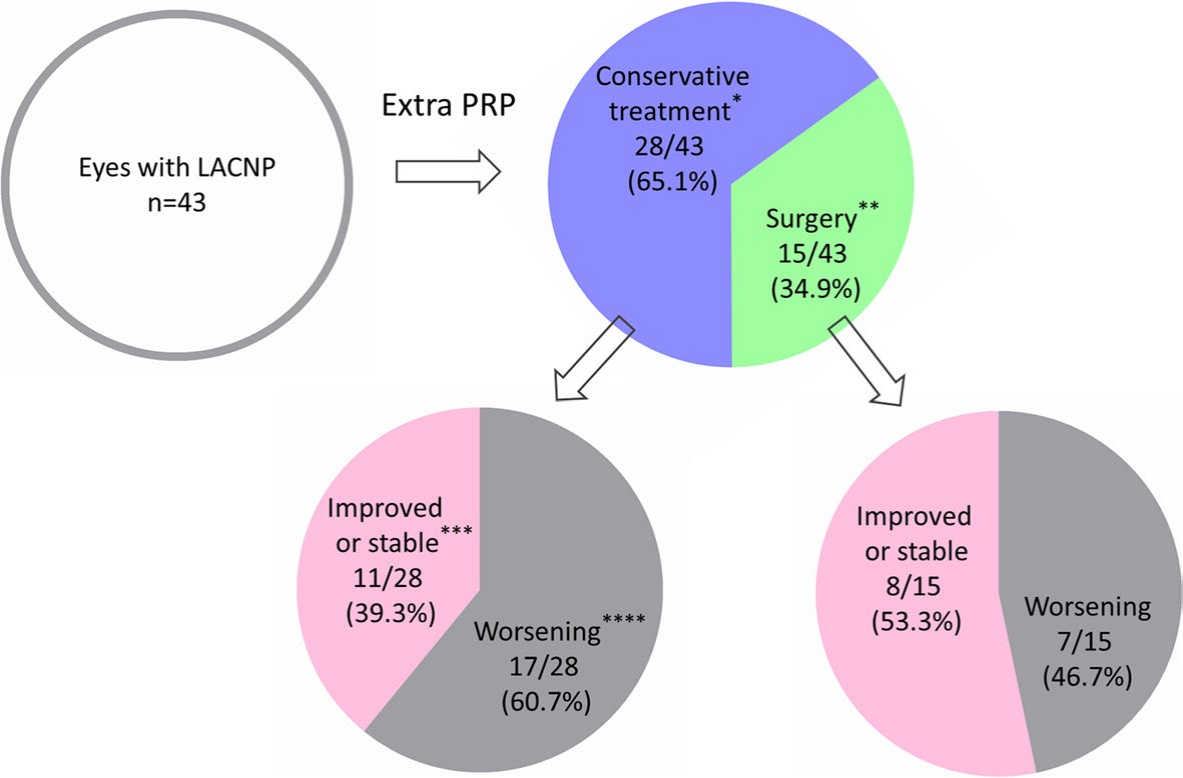
A diagram showing treatment intervention and prognosis in eyes with large area of capillary non-perfusion (LACNP). ^*^Conservative medical treatment was applied in eyes with no evidence of neovascular complications (*n*=23) or with end-stage of diabetic retinopathy indicating very poor surgical prognosis (*n*=5). ^**^Surgical intervention (*n*=15) included pars plana vitrectomy for vitreous hemorrhage and/or tractional detachment (*n*=11) and anti-glaucoma surgery (*n*=4). ^***^Improved or stable was defined as an increase of BCVA or a decrease of less than one ETDRS line (5 letters) without significant neovascular complications. ^****^Worsening was considered with a decrease of 1 EDTRS line or more. PRP: pan retinal photocoagulation

The mean follow-up duration was 12.4 ± 16.7 months. During this period, 20 eyes (46.5%) demonstrated advanced stage of proliferative DR, including vitreous hemorrhage, tractional retinal detachment or neovascular glaucoma. Eleven of these eyes received PPV with (*n*=10) or without (*n*=1) phacoemulsification, of which 8 eyes had their BCVA improved or maintained until the last follow-up with no evidence of DR progression whereas 3 eyes showed deteriorated BCVA with progression of DR. Another 4 eyes underwent anti-glaucoma surgery, all of which showed poor visual function in the last follow-up. Conservative medical treatment was selected in the rest eyes due to end-stage of DR indicating a poor surgical prognosis (*n*=5) or absence of severe neovascular complications (*n*=23). Eleven of these non-operated eyes show no or mild progression of DR with maintained visual acuity while the other 17 eyes developed varying degrees of worsening of BCVA and DR (Fig. 3, Table 3). Overall, in the final follow-up, BCVA improved or maintained stable in 19 eyes (44.2%) and deteriorated in 24 eyes (55.8%).

**Table 3:** Treatment and outcomes in eyes with LACNP

	Number (percentage)
Laser burns in PRP	1798.0 ± 724.9
Macular grid laser	5 (11.6%)
Follow-up duration (months)	12.4 ± 16.7
logMAR BCVA	
Improved	12 (27.9%)
Stable	7 (16.3%)
Deteriorated	24 (55.8%)
Complications	
VH	12 (27.9%)
TRD	6 (14.0%)
NVG	9 (20.9%)
Intervention	
Anti-VEGF agents	17 (39.5%)
PPV surgery	11 (25.6%)
Anti-glaucoma surgery	4 (9.3%)

*Abbreviations*: *PRP* pan-retinal photocoagulation, *BCVA* best corrected visual acuity, *FFA* fluorescence fundus angiography, *VEGF* vascular endothelial growth factor, *PPV* pars plana vitrectomy, *VH* Vitreous hemorrhage, *TRD* Tractional retinal detachment, *NVG* Neovascular glaucoma. Data are mean ± SD

**Fig. 4:**
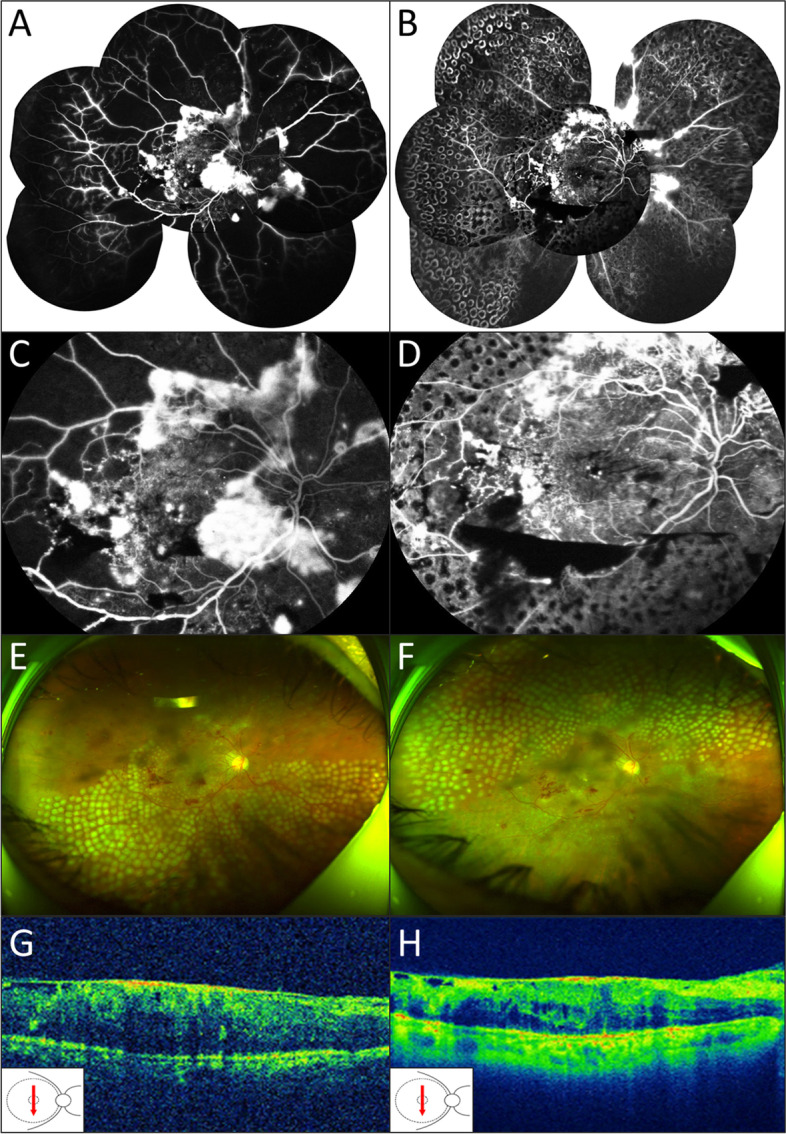
Representative images of a patient with LACNP who presented stable visual acuity and mild progression of DR during the one-year follow-ups. Angiographic images showing LACNP at his first presentation (**A**) and 1 year after non-surgical treatment (**B**). **C**-**D** Angiographic images showing detailed information of the posterior pole before and after treatment. At first time of FFA, macular capillary perfusion combined with multiple vascular leakages was seen (**C**). Macular capillary perfusion was maintained one year after treatment. Vascular leakage was reduced while local hemorrhage could be seen (**D**). **E**-**F** Scanning laser ophthalmoscopy showing pan-retinal photocoagulation divided into 2 sessions. **G**-**H** Optical coherence tomography revealed diffused macular edema and local epimacular membrane at the first presentation. The central macular thickness was 548 μm before extensive PRP (**G**). At the final follow-up after treatment, macular edema showed mild reduction with the central macular thickness of 502 μm (**H**)

**Fig. 5:**
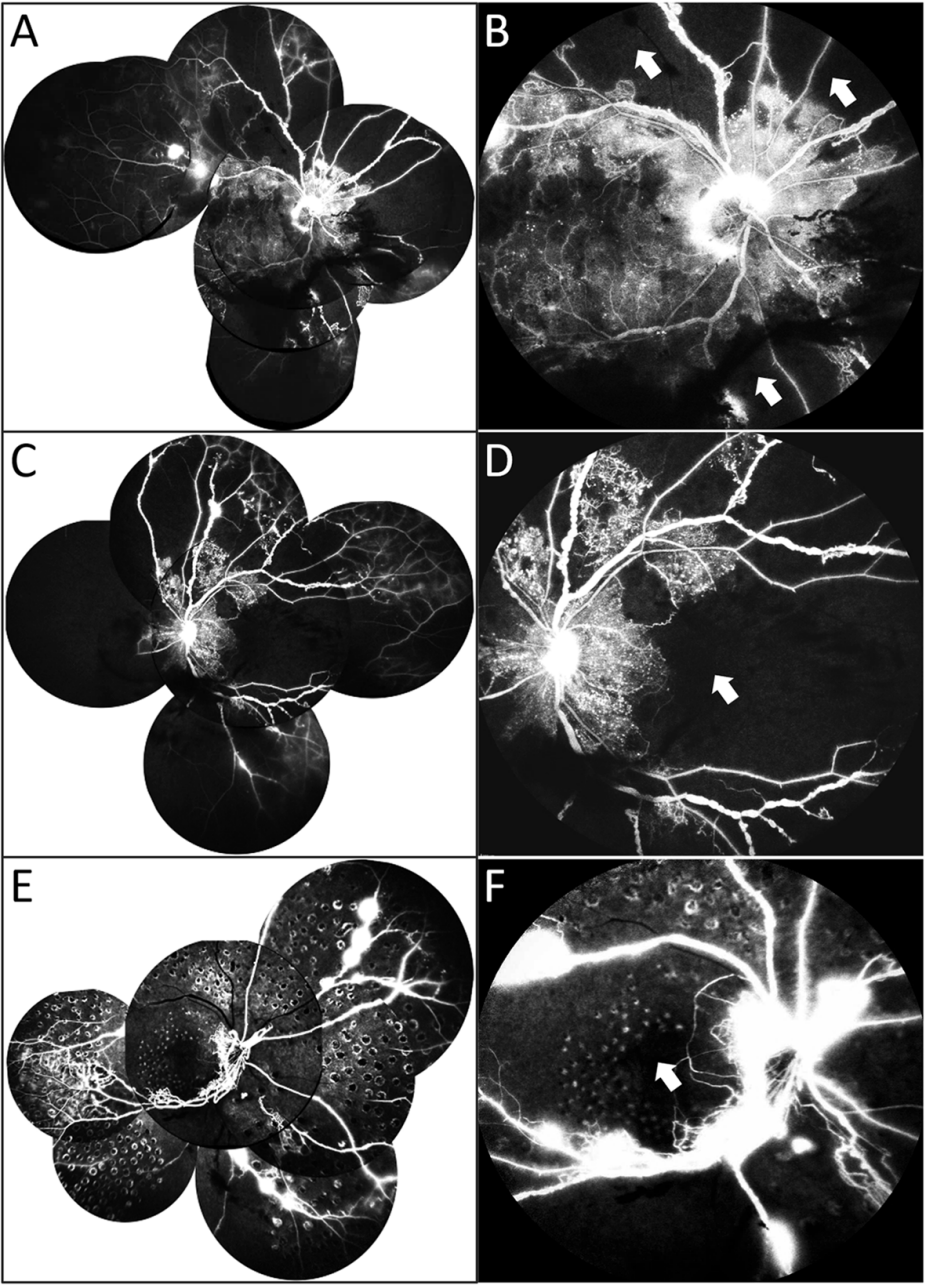
Representative images of a patient with LACNP who developed rapid worsening of DR within a few months. **A**-**B** At first presentation, LACNP was found in the peripheral retina except the macula region in his right eye (Arrow indicated peripheral non-perfusion area in panel **B**). **C**-**D** In his left eye, FFA exam revealed large area of capillary non-perfusion spreading the whole retina before treatment (Arrow indicated macular non-perfusion in panel **D**). **E**-**F** He received extensive pan-retinal laser photocoagulation in his right eye but still developed macular non-perfusion several months later with very poor visual acuity (Arrow indicated macula-involved non-perfusion in panel **F**)

Some patients could have their central visual acuity preserved for a relatively long time with proper treatment. A 56-year-old male with bilateral LACNP showed the BCVA of 20/200 in his right eye. He received extensive PRP with proper glycemic control. After 1 year, BCVA maintained as 20/200 with no visible worsening of CNP (Fig. 4). In his left eye, PPV with supplementary laser photocoagulation was performed due to vitreous hemorrhage. BCVA improved to 20/125 and IOP was normal in the 1-year follow-up compared with that of 20/400 before surgical treatment. However, the presence of LACNP more often indicated poor prognosis. A 44-year-old male presented bilateral LACNP The initial BCVA was 20/33 OD and 20/66 OS. He received bilateral extensive PRP but had poorly-controlled diabetes and hypertension. Several months later, he developed macular CNP in his right eye with poor visual acuity (20/400). He also suffered from neovascular glaucoma and vitreous hemorrhage with an IOP of 57 mmHg in his left eye and underwent Ahmed valve implantation. IOP was well-controlled whereas visual acuity dropped to no light perception (Fig. 5).

## Discussion

As an important characteristic of DR, the area of capillary non-perfusion (CNP) has been studied for years. However, according to previous literatures, the percentage of CNP in DR population mainly ranged from 25%-50% and little is reported on extensively large area of capillary non-perfusion [8, 9]. In this study, based on FFA images, LACNP was defined as over 70% of CNP area through the total visible retina. To the best of our knowledge, this is a pilot study focusing on DR with LACNP in a larger clinical sample, aiming to describe its characteristics, treatments and prognosis.

The area of CNP serves as a potential useful biomarker for severity and proliferative activity of DR [5, 6]. As compared with mild NPDR, the moderate to severe NPDR group had 2.7 lower and the PDR group had 4.3 lower perfusion index (percent coverage of area by retinal vessels with flow), indicating an inverse association between perfusion index and DR severity [6]. In addition, Lee et al. found the area of peripheral CNP to be significantly larger in patients with active PDR than those who failed to develop PDR 6 months after laser treatment [5]. An association between cystoid macular edema and untreated peripheral non-perfusion was also demonstrated [11]. These studies indicate the importance of CNP assessment in predicting the severity and progression of DR. Measurements of the area of CNP may be helpful to differentiate patients who are at high risk for the development of neovascularization and proliferation.

The potential risk factors for LACNP remain unclear, which may include the course of diabetes, levels of blood glucose and HbAlc, hypertension and arteriosclerosis, prior intravitreal treatment of anti-VEGF agents, etc [12]. In this study, the presenting diabetes duration was found to be 7.3 years on average, which seemingly had no difference with that of general DR populations. However, a longer DM duration may exist in these patients because of undiscovered diabetes at its early stage since less attention was given to annual physical exam in rural China. As reported, most retinopathy and nephropathy occur over 10 years after onset of diabetes according to the disease guidelines [13–15]. No treatment during the early stage of diabetes may lead rapid worsening of DR. In addition, poor control of diabetes also contributes to the development of LACNP. The fasting blood glucose and HbAlc levels in this group, under medical treatment, were 13.5 mmol/L and 8.2% on average at first presentation, indicating the poor control in the past time. It was also noted that 75.0% of patients with LACNP exhibited elevated levels of creatinine, urea nitrogen, and that 37.5% of them suffered from hypertension. These data indicate that the LACNP may act as a predictive marker for advanced diabetes mellitus with long duration and a lack of proper treatment. Future study is needed to determine the role of these risk factors as well as others such as gender, age, a history of smoking, and cardiovascular event, in patients with LACNP.

The occurrence rate of LACNP was 3.3% in this study, which accounted for a considerable percentage in DR population. One possible reason for the high detection rate is that we conducted FFA routinely for DR population as it provides more information than single fundus photography [16]. It was found that patients with LACNP may present moderate DR on fundus photograph showing only microaneurysm, splinter hemorrhage and exudation, which can hardly be distinguished from a mild NPDR (Supplementary Figure 1). These patients, if left untreated or improperly managed, may progress rapidly to advanced DR with poor visual function. Our data highlights the necessity and importance of FFA in evaluating the severity of DR, especially in young patients with poorly-controlled diabetes and hypertension, in order to access accurate diagnosis and prompt and effective treatment.

Based on the distribution of retinal capillaries, the CNP usually starts at the equator with lower oxygen supply and then extends towards the peripheral region with DR progression [17]. The macular is nourished by multilayers of vascular plexus [18]. The presence of LACNP in the macular area therefore indicates very severe ischemia. Based on our existing data, patients younger than 50-year-old with poorly-controlled diabetes and hypertension are likely to suffer from rapid development of macular CNP and poor visual outcomes within months to a year.

The presence of LACNP usually predicts poor visual prognosis. In this study, 20 eyes (46.5%) developed vitreous hemorrhage, tractional retinal detachment or neovascular glaucoma during the follow-up periods despite extensive laser photocoagulation and proper control of systemic diseases and 24 eyes (55.8%) had their final BCVA deteriorated from 1.0 ± 0.6 to 1.5 ± 0.6 logMAR, indicating the worsening natural course in eyes even though ocular and systemic treatment was taken. In addition, nearly half of the patients (47.4%) suffered from bilateral vision loss, further indicating the importance of early DR screening and treatments.

There have been no treatment guidelines for LACNP. Laser photocoagulation has been used for decades as the main treatment strategy in DR [19]. A pan-retinal photocoagulation (PRP) can largely slow down the growth and development of new blood vessels and prevent the subsequent neovascular complications [20]. However, when dealing with LACNP, standard PRP treatment may become insufficient to prevent neovascular complications. In this study, prompt and extensive retinal laser photocoagulation was applied for all eyes with LACNP, in which the burns were separated by a small gap and extended as close to the ora serrata. In addition, the type, pattern, and density of laser photocoagulation may have varying effects on an eye with PDR. The 532nm laser used in this study has been proved to be effective for PDR in the regression of neovascularization as well as the resolution of retinopathy, although other lasers such as pattern scan laser (PASCAL) may be potentially less time consuming and less painful [21]. Previous studies have also suggested the combination of anti-VEGF agents and PRP to treat PDR without significant macular edema, with regards to induce neovascular regression and reduce the risk of macular edema after PRP [22, 23]. In addition, it remains unclear whether anti-VEGF will cause further microvascular degeneration and regression in eyes already with extensive non-perfusion [24, 25]. The effectiveness of combination therapy for LACNP deserves further investigation.

Surgical intervention should be considered when neovascular complications occur. In this study, 11 eyes received PPV with (*n*=10) or without (*n*=1) phacoemulsification for proliferative DR and 4 eyes underwent anti-glaucoma surgery for refractory neovascular glaucoma. Intravitreal injection was performed prior of surgery in all of these eyes to prevent intraoperative and early postoperative hemorrhage, as well as further neovascularization. Of note, 8 in 11 eyes receiving anti-VEGF agents and PPV surgery had the visual function improved or maintained with no evidence of DR worsening at a mean follow-up time of 21.0 months. The other 3 eyes showed slight vision decrease without progressing to neovascular glaucoma. These data suggest that aggressive therapy may slow DR progression by preserving macular microperfusion and therefore maintain central vision for some time.

In this study, all patients were asked for intensive antidiabetic and antihypertensive treatment in general hospitals, which we believed played a role in slowing the progression of DR. The association between early worsening of DR with poorly controlled systemic diseases has been demonstrated [26]. A meta-analysis study in patients with type 2 diabetes suggest that intensive glucose lowering, compared with the conventional group, exhibited a 13% risk reduction in ocular events after 5 years of follow-up, including the requirement for PPV surgery, development of PDR and progression of DR [27]. Poorly controlled hypertension is another potential risk factor. Evidence-based studies suggest an optimal blood pressure of lower than 130/80 mmHg in diabetic populations [28]. In this study, approximately 80% of the patients suffered from bilateral LACNP, indicating the impact of systemic conditions. In addition, blood glucose critical values and glycemic excursion were monitored for multiple times in hospital despite insulin treatment, implicating very poor control of diabetes. Finally, it was noted that patients who had their diabetes and hypertension well-controlled within an acceptable range tended to had stabilized visual function and slow progressing retinopathy, further indicating the important role of intensive control of systemic disease in preventing the progression of DR.

The ultra-widefield fluorescein angiographic system has been described in recent years, which owns its superiority in enabling the imaging of up to 200° of the posterior pole in a single image, compared with that of 30-55° per image in conventional FFA [29, 30]. However, the penetration rate of this system is low in many medical institutions. In addition, the wide-angle system leads to distortion in the size and scale of the central and peripheral region, making the measurement inaccurate [29, 30]. Conversely, conventional FFA is very popular, provides higher resolution images with less distortion, and ensures more precise measurement. In this study, we used spliced conventional FFA images to evaluate the area of CNP, which showed limited image retina (about 130° of the posterior pole). Although the peripheral retina was supposed to be non-perfused since only main blood vessels in the mid-periphery were observed, the possibility of capillary perfusion in the very peripheral area could not be ruled out. Based on the distribution of retinal capillaries, the CNP usually starts at the equator and extends towards the peripheral region. The macula, nourished by multilayers of capillary network, showed better tolerance to ischemia insults [31]. In this study, 25 eyes (58.1%) presented with macular ischemia with poor visual acuity at their first presentation, indicating the presence of end-stage DR. Further research in this field using wide-field angiography is valuable to compare the CNP results from this study.

The present study is limited by the retrospective design, small sample size, and the lack of a control group. Due to the retrospective design, it is difficult to focus on factors that may have had some impact on the visual acuity outcomes, such as the development of cataract and macular edema, and retinal structural changes, although the microperfusion changes of the macula, which mainly influence the visual outcomes, has been observed. It remains also challenging to determine whether and when patients with LACNP develop to advanced stages of DR, whether intensive treatments can prevent LACNP from worsening, and what could be the optimal treatment strategies for these patients.

## Conclusions

We describe the imaging characteristics and prognosis of a subgroup of patients with DR who present large area of capillary non-perfusion. The LACNP may associate with long duration and poorly-controlled diabetes and hypertension, predicts rapid worsening of DR, and acts as a signal for advanced diabetes with an unfavorable visual prognosis. Early detection and prompt treatments including extensive laser photocoagulation and surgical interventions are recommended, which may potentially slow DR progression and maintain central vision for some time. In addition, we recommend early FFA exam in patients with DR which is necessary for accurate visualization and assessment of retinal microperfusion. Finally, wide-field angiography, with its increasing popularity in future days, may be useful for a better understanding of this phenomenon.

## Supplementary Information

**Additional file 1.**

## Data Availability

All data supporting these findings are contained within this manuscript.

## Abbreviations

BCVA: best-corrected visual acuity
CNP: capillary non-perfusion
DR: diabetic retinopathy
DRIL: disorganization of retinal inner layers
EZ: ellipsoid zone
FFA: fluorescence fundus angiography
LACNP: large area of capillary non-perfusion
NPDR: non-proliferative diabetic retinopathy
OCT: optical coherence tomography
PDR: proliferative diabetic retinopathy
PPV: pars plana vitrectomy
PRP: pan-retinal laser photocoagulation
SLO: scanning laser ophthalmoscope
VEGF: vascular endothelial growth factor

## References

[1] Cheung N, Mitchell P, Wong TY (2010). Diabetic retinopathy. Lancet.

[2] Owen LA, Hartnett ME (2013). Soluble mediators of diabetic macular edema: the diagnostic role of aqueous VEGF and cytokine levels in diabetic macular edema. Curr Diab Rep.

[3] Capitão M, Soares R (2016). Angiogenesis and Inflammation Crosstalk in Diabetic Retinopathy. J Cell Biochem.

[4] Vinores SA, Youssri AI, Luna JD, Chen YS, Bhargave S, Vinores MA, Schoenfeld CL, Peng B, Chan CC, LaRochelle W (1997). Upregulation of vascular endothelial growth factor in ischemic and non-ischemic human and experimental retinal disease. Histol Histopathol.

[5] Torp TL, Kawasaki R, Wong TY, Peto T, Grauslund J (2019). Peripheral capillary non-perfusion in treatment-naive proliferative diabetic retinopathy associates with postoperative disease activity 6 months after panretinal photocoagulation. Br J Ophthalmol.

[6] Lin AD, Lee AY, Zhang Q, Rezaei KA, Kinyoun J, Wang RK, Lee CS (2017). Association between OCT-based microangiography perfusion indices and diabetic retinopathy severity. Br J Ophthalmol.

[7] Ehlers JP, Jiang AC, Boss JD, Hu M, Figueiredo N, Babiuch A, Talcott K, Sharma S, Hach J, Le T (2019). Quantitative Ultra-Widefield Angiography and Diabetic Retinopathy Severity: An Assessment of Panretinal Leakage Index, Ischemic Index and Microaneurysm. Count Ophthalmology.

[8] Rasta SH, Nikfarjam S, Javadzadeh A (2015). Detection of retinal capillary nonperfusion in fundus fluorescein angiogram of diabetic retinopathy. Bioimpacts.

[9] Zheng Y, Kwong MT, Maccormick IJ, Beare NA, Harding SP (2014). A comprehensive texture segmentation framework for segmentation of capillary non-perfusion regions in fundus fluorescein angiograms. PLoS One.

[10] Flaxel CJ, Adelman RA, Bailey ST, Fawzi A, Lim JI, Vemulakonda GA, Ying GS (2020). Diabetic Retinopathy Preferred Practice Pattern(R). Ophthalmology.

[11] Oliver SC, Schwartz SD (2010). Peripheral vessel leakage (PVL): a new angiographic finding in diabetic retinopathy identified with ultra wide-field fluorescein angiography. Semin Ophthalmol.

[12] Atchison E, Barkmeier A (2016). The Role of Systemic Risk Factors in Diabetic Retinopathy. Curr Ophthalmol Rep.

[13] Klein R, Klein BE, Moss SE, Davis MD, DeMets DL (1984). The Wisconsin epidemiologic study of diabetic retinopathy. II. Prevalence and risk of diabetic retinopathy when age at diagnosis is less than 30 years. Arch Ophthalmol.

[14] Varma R, Torres M, Pena F, Klein R, Azen SP, Los Angeles Latino Eye Study G (2004). Prevalence of diabetic retinopathy in adult Latinos: the Los Angeles Latino eye study. Ophthalmology.

[15] Svensson M, Sundkvist G, Arnqvist HJ, Björk E, Blohmé G, Bolinder J, Henricsson M, Nyström L, Torffvit O, Waernbaum I (2003). Signs of nephropathy may occur early in young adults with diabetes despite modern diabetes management: results from the nationwide population-based Diabetes Incidence Study in Sweden (DISS). Diabetes Care.

[16] Wang S, Zuo Y, Wang N, Tong B (2017). Fundus fluorescence Angiography in diagnosing diabetic retinopathy. Pak J Med Sci.

[17] Yu DY, Cringle SJ, Yu PK, Balaratnasingam C, Mehnert A, Sarunic MV, An D, Su EN (2019). Retinal capillary perfusion: Spatial and temporal heterogeneity. Prog Retin Eye Res.

[18] Provis JM, Penfold PL, Cornish EE, Sandercoe TM, Madigan MC (2005). Anatomy and development of the macula: specialisation and the vulnerability to macular degeneration. Clin Exp Optom.

[19] Palanker DV, Blumenkranz MS, Marmor MF (2011). Fifty years of ophthalmic laser therapy. Arch Ophthalmol.

[20] Photocoagulation treatment of proliferative diabetic retinopathy: the second report of diabetic retinopathy study findings. Ophthalmology 1978, 85(1):82-106. 10.1016/s0161-6420(78)35693-1 345173

[21] Nagpal M, Marlecha S, Nagpal K (2010). Comparison of laser photocoagulation for diabetic retinopathy using 532-nm standard laser versus multispot pattern scan laser. Retina.

[22] Filho JA, Messias A, Almeida FP, Ribeiro JA, Costa RA, Scott IU, Jorge R (2011). Panretinal photocoagulation (PRP) versus PRP plus intravitreal ranibizumab for high-risk proliferative diabetic retinopathy. Acta Ophthalmol.

[23] Cho WB, Oh SB, Moon JW, Kim HC (2009). Panretinal photocoagulation combined with intravitreal bevacizumab in high-risk proliferative diabetic retinopathy. Retina.

[24] Chung EJ, Roh MI, Kwon OW, Koh HJ (2008). Effects of macular ischemia on the outcome of intravitreal bevacizumab therapy for diabetic macular edema. Retina.

[25] Munk MR, Ceklic L, Ebneter A, Huf W, Wolf S, Zinkernagel MS (2016). Macular atrophy in patients with long-term anti-VEGF treatment for neovascular age-related macular degeneration. Acta Ophthalmol.

[26] Wat N, Wong RL, Wong IY (2016). Associations between diabetic retinopathy and systemic risk factors. Hong Kong Med J.

[27] Zoungas S, Arima H, Gerstein HC, Holman RR, Woodward M, Reaven P, Hayward RA, Craven T, Coleman RL, Chalmers J (2017). Effects of intensive glucose control on microvascular outcomes in patients with type 2 diabetes: a meta-analysis of individual participant data from randomised controlled trials. Lancet Diabetes Endocrinol.

[28] Zoungas S, de Galan BE, Ninomiya T, Grobbee D, Hamet P, Heller S, MacMahon S, Marre M, Neal B, Patel A (2009). Combined effects of routine blood pressure lowering and intensive glucose control on macrovascular and microvascular outcomes in patients with type 2 diabetes: New results from the ADVANCE trial. Diabetes Care.

[29] Wessel MM, Aaker GD, Parlitsis G, Cho M, D'Amico DJ, Kiss S (2012). Ultra-wide-field angiography improves the detection and classification of diabetic retinopathy. Retina.

[30] Rabiolo A, Parravano M, Querques L, Cicinelli MV, Carnevali A, Sacconi R, et al. Ultra-wide-field fluorescein angiography in diabetic retinopathy: a narrative review. Clin Ophthalmol. 2017;11:803–7. 10.2147/OPTH.S133637. 10.2147/OPTH.S133637 PMC5415004 28490862

[31] Lavia C, Mecê P, Nassisi M, Bonnin S, Marie-Louise J, Couturier A, Erginay A, Tadayoni R, Gaudric A (2020). Retinal Capillary Plexus Pattern and Density from Fovea to Periphery Measured in Healthy Eyes with Swept-Source Optical Coherence Tomography Angiography. Sci Rep.

