# Supplementary figures and images for: Diabetic retinopathy with extensively large area of capillary non-perfusion: characteristics and treatment outcomes
Source: BMC Ophthalmol. 2022 Jul 4;22:293. doi: 10.1186/s12886-022-02508-6 (PMC9254521; doi:10.1186/s12886-022-02508-6)

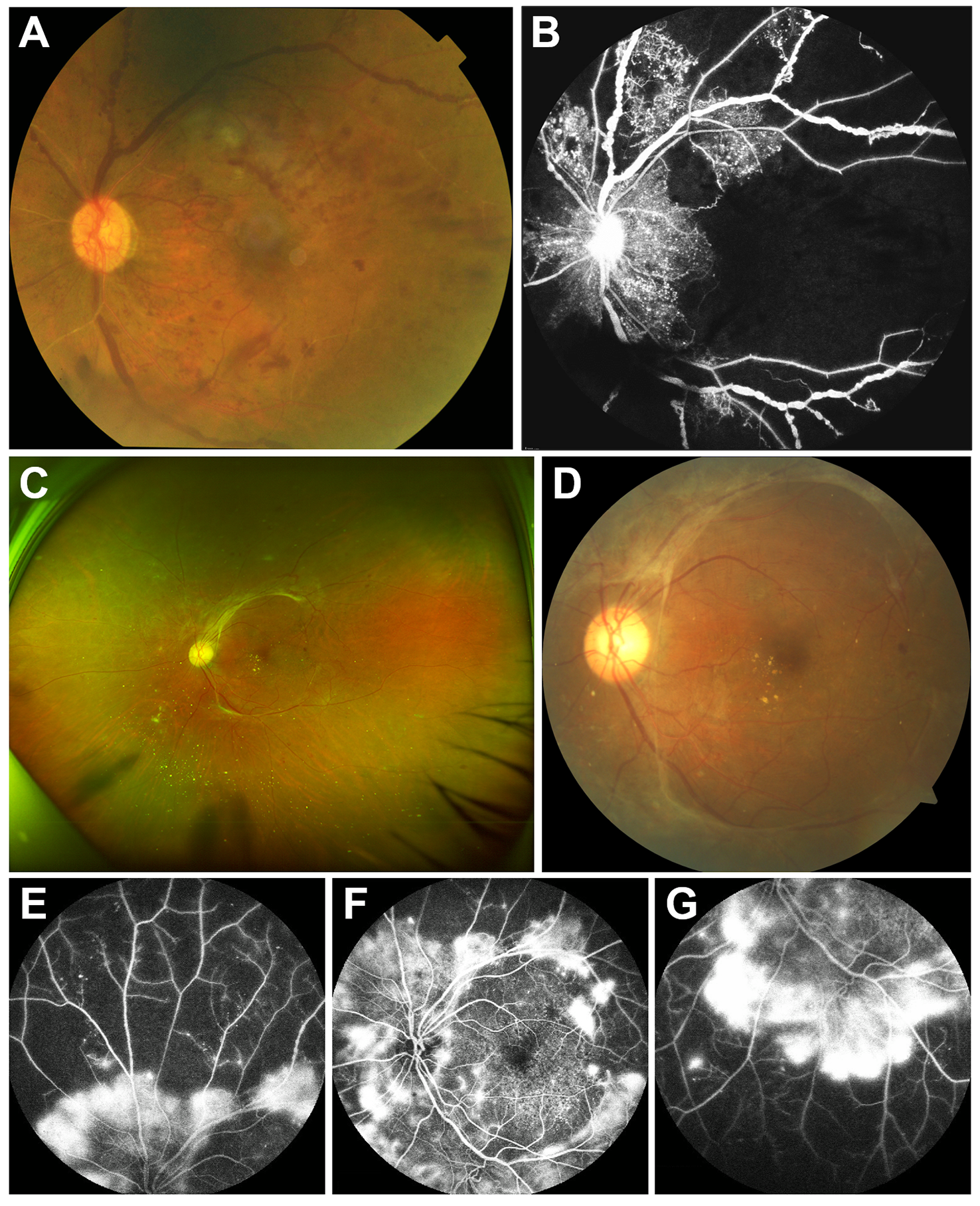

Supplement: Supplementary file 1 — Additional file 1. [file 12886_2022_2508_MOESM1_ESM.tif]
